# Effects of HIV-1 genotype on baseline CD4+ cell count and mortality before and after antiretroviral therapy

**DOI:** 10.1038/s41598-020-72701-4

**Published:** 2020-09-28

**Authors:** Zhiqiang Cao, Jianjun Li, Huanhuan Chen, Chang Song, Zhiyong Shen, Xinjuan Zhou, Guanghua Lan, Qiuying Zhu, Shujia Liang, Hui Xing, Lingjie Liao, Yi Feng, Yiming Shao, Yuhua Ruan

**Affiliations:** 1 State Key Laboratory of Infectious Disease Prevention and Control (SKLID), National Center for AIDS/STD Control and Prevention (NCAIDS), Chinese Center for Disease Control and Prevention (China CDC), Collaborative Innovation Center for Diagnosis and Treatment of Infectious Diseases, Beijing, China; 2 Guangxi Zhuang Autonomous Region Center for Disease Prevention and Control, Nanning, China

**Keywords:** Computational biology and bioinformatics, Medical research, Risk factors

## Abstract

To assess whether human immunodeficiency virus type 1 (HIV-1) genotype influences baseline CD4+ T lymphocyte (CD4+) cell count and mortality of patients. The study was conducted from 2014 to 2019 in Guangxi, China, and included 2845 newly diagnosed HIV patients. We used a median regression model to compare CD4+ cell counts in patients newly diagnosed with different HIV-1 genotypes, and a Cox regression model to analyze the associations between HIV-1 genotypes and mortality before and after antiretroviral treatment (ART). In newly diagnosed HIV patients, the baseline CD4+ cell counts of patients with CRF01_AE were significantly lower than those of patients with CRF07_BC, CRF08_BC, and other genotypes. Compared with CRF01_AE, patients infected with CRF07_BC (hazard ratio, 0.55; 95% CI 0.36–0.85), CRF08_BC (hazard ratio, 0.67; 95% CI 0.52–0.85), or other genotypes (hazard ratio, 0.52; 95% CI 0.29–0.94) had significantly lower mortality rates before ART. There were no significant associations between different HIV-1 genotypes and mortality after ART. HIV-1 genotype significantly influences baseline CD4+ cell count and mortality before ART in newly diagnosed HIV patients. We find no significant difference in the outcome of death after ART in patients with different HIV-1 genotypes.

## Introduction

Human immunodeficiency virus (HIV) exhibits a broad range of genetic heterogeneity^1^. The HIV epidemic is mainly caused by HIV-1 group M, which accounts for at least 90% of HIV infections worldwide^2^. HIV-1 group M is further divided into distinct genotypes, namely A, B, C, D, F, G, H, J, and K, as well as hybrids resulting from recombination between genotypes, known as circulating recombinant forms (CRFs) or unique recombinant forms (URFs)^2,3^. This genetic diversity is a major obstacle to the development of a vaccine against HIV, as an effective vaccine must protect against diverse HIV genotypes^4^. Furthermore, HIV genetic diversity may also affect virus pathogenicity, disease progression, and response to antiretroviral treatment (ART) among infected patients^1,5^. A relatively consistent finding is that compared with genotype A, genotype D is associated with mortality before ART^6–8^. Research on HIV-1 subtypes and ART showed that infection with genotype A results in increased mortality compared to that with genotype B in Europe and Canada^9^. In China, studies showed that CRF01_AE is associated with faster disease progression from estimated seroconversion to acquired immune deficiency syndrome (AIDS) compared with non-CRF01_AE in ART-naïve patients^10,11^. These aforementioned studies revealed that HIV-1 genotype differences contribute to variations in disease progression and ART efficiency. However, studies focused on CRF01_AE featured small participant numbers and short follow-up periods, which necessitates broader comparisons between one genotype and all other genotypes grouped together. Furthermore, only a few studies have focused on the CRF07_BC and CRF08_BC genotypes, which are prevalent in China^12–14^. In addition, the effects of CRF01_AE, CRF07_BC, and CRF08_BC have not been directly assessed using mortality as a study end point for either ART-naïve participants or those on ART. This question is urgent with respect to HIV-1 epidemic in China, as transmission accounts for more than 90% of cases, resulting in infections by CRF07_BC, CRF08_BC, and multilineages of CRF01_AE; this is distinct from the single lineage epidemic of B genotype prevalent in European countries^12,13,15,16^. These genotypes are also increasing rapidly among the global proportion of genotypes over time^1,2^.

At the end of 2017, more than 70,000 infected patients were living with HIV/AIDS in Guangxi, one of the most important epicenters of the HIV epidemic in China^17^. Previous studies have shown that the cumulative mortality of HIV in Guangxi could reach three times that of the national average and up to nine times that of some developed countries, which is likely associated with the late discovery of HIV infection^18–22^. In recent years, studies have revealed that the prevalence of HIV-1 CRF01_AE, CRF07_BC, and CRF08_BC is expanding rapidly in southwestern China^13^. Assessing genotypes as risk factors for disease progression and the response to ART is both urgent and necessary for better understanding the virus characteristics, justifying the early initiation and efficiency of ART, and reducing mortality and transmission rates. This study was performed to investigate the effects of HIV-1 genotype on baseline CD4+ cell counts and mortality, before and after ART, among newly diagnosed HIV patients. We found that CRF01_AE was significantly associated with a lower baseline CD4+ count and higher mortality before ART treatment, and that combination ART treatment is equally efficient among HIV patients regardless of their HIV-1 genotype.

## Results

### Baseline characteristics of study participants

**Figure 1:**
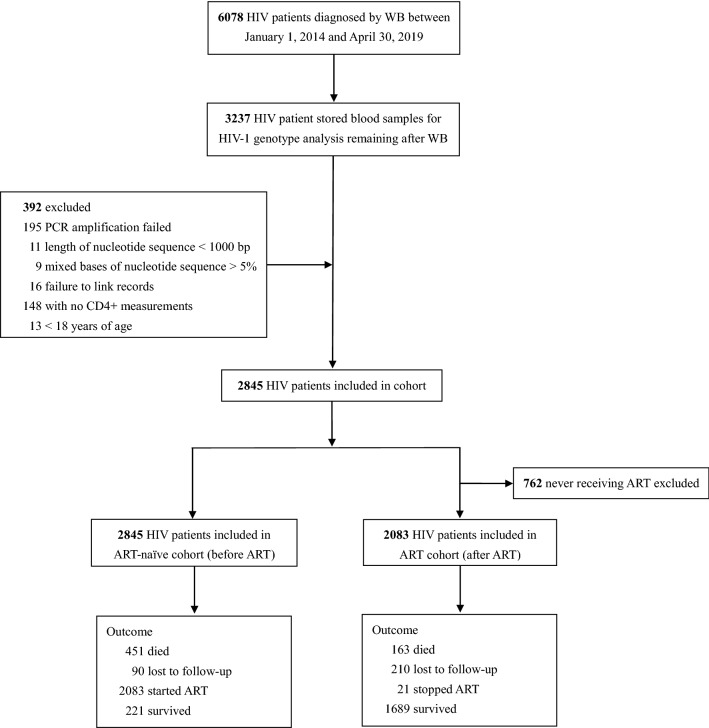
Flow diagram of study. *ART* antiretroviral therapy, *WB* western blotting. We performed this observational cohort study using data from the HIV/AIDS Comprehensive Response Information Management System (CRIMS) in Guangxi, China. Local health workers completed standardized reporting forms recording baseline information when HIV patients were newly diagnosed, and follow-up visits then conducted.

Of the 6,078 HIV patients who were newly diagnosed between January 1, 2014 and April 30, 2019, 3237 had blood samples available in storage for HIV-1 genotype analysis after western blotting (WB) confirmation tests. Some patients (n = 392) were excluded due to nested polymerase chain reactions (PCR) amplification failure (n = 195), length of the nucleotide sequence being less than 1000 base pairs (n = 11), mixed bases of nucleotide sequence being more than 5% (n = 9), failure to link records (n = 16), no CD4+ T lymphocyte (CD4+) cell count measurements (n = 148), or being under the age of 18 years (n = 13). Among 2845 patients who met the eligibility criteria, 2845 HIV patients entered the ART-naïve cohort (before ART) and 2083 HIV patients were included in the ART cohort (after ART; Fig. 1).

**Figure 2:**
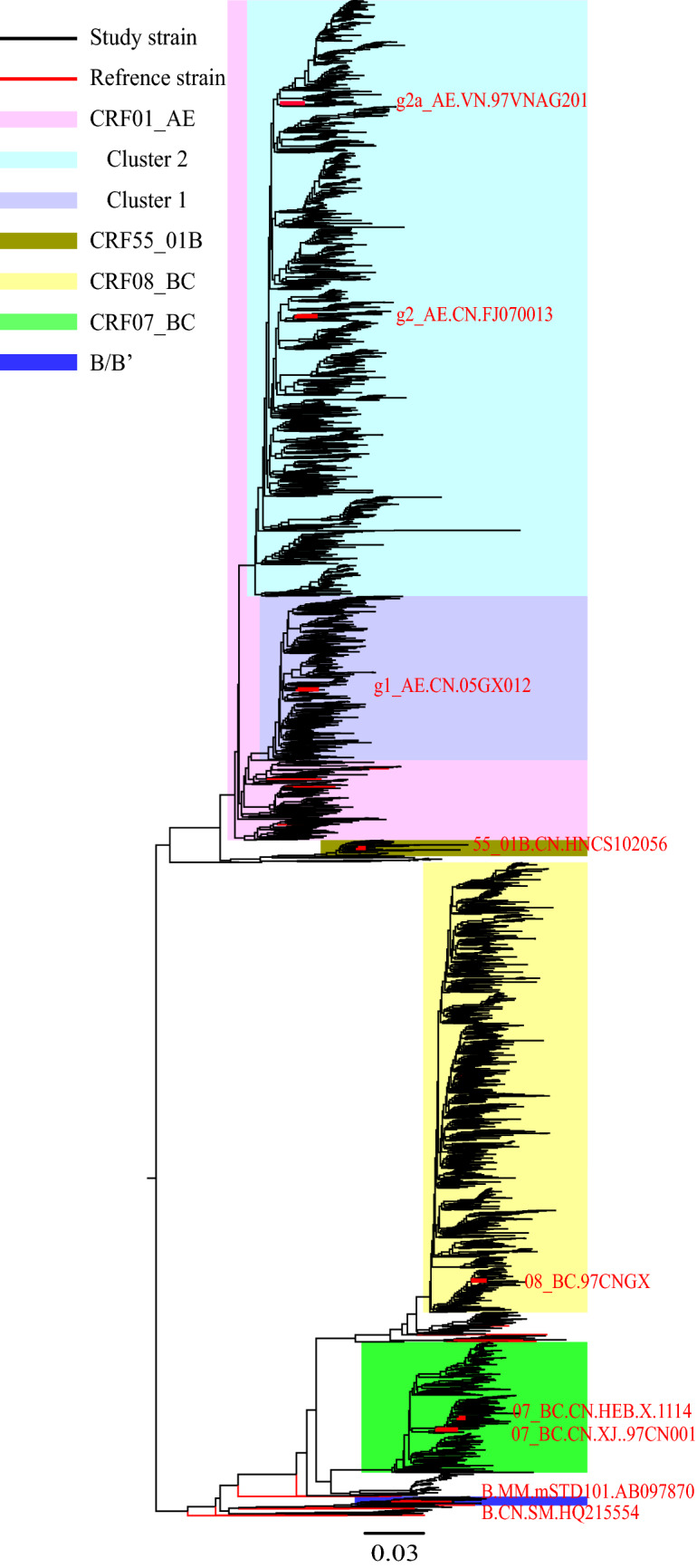
Phylogenetic tree of HIV-1 *Pol.* Shimodaira–Hasegawa test was used to alternate the topologies of the whole sequences. Branches, representing their respective sequences, forming node with high confidence (> 90%) was defined as the same type of genotype. The scale length (range: 0–1) represents the difference between sequences (range: 0–100%).

The baseline characteristics of the 2845 patients are shown in Table 1. Most patients were over 50 years of age (53.1%), male (74.2%), married or cohabiting (60.5%), Han (81.5%), educated up to primary school level or less (55.3%), employed in farming (78.8%), and infected through heterosexual contact (94.2%). The median time from HIV-positive diagnosis to baseline CD4+ cell count measurements was 7 days [interquartile range (IQR), 2–23 days; Table 1]. A phylogenetic genotype tree is shown in Fig. 2. The baseline genotyping data of the 2845 study participants revealed that the predominant genotype was CRF01_AE for 1687 patients (59.3%; 1221 with CRF01_AE Cluster 2, 331 with CRF01_AE Cluster 1, and 135 with other clusters), followed by CRF08_BC for 728 patients (25.6%), CRF07_BC for 254 patients (8.9%), and other genotypes for 176 patients (6.2%; 59 with various other CRFs, 68 with URFs, 37 with CRF55_01B, and 12 with B/B’).

**Table 1:** Baseline characteristics of study participants.

Variable	Total	HIV-1 genotype				P
		CRF01_AE	CRF07_BC	CRF08_BC	Others^#^	
	N (%)	N (%)	N (%)	N (%)	N (%)	
Total	2845 (100.0)	1687 (59.3)	254 (8.9)	728 (25.6)	176 (6.2)	
**Age at diagnosis, years**						
18–29	279 (9.8)	143 (8.5)	49 (19.3)	57 (7.8)	30 (17.0)	< 0.001
30–49	1054 (37.1)	593 (35.1)	62 (24.4)	318 (43.7)	81 (46.1)	
≥ 50	1512 (53.1)	951 (56.4)	143 (56.3)	353 (48.5)	65 (36.9)	
**Sex**						0.001
Male	2112 (74.2)	1257 (74.5)	200 (78.7)	510 (70.1)	145 (82.4)	
Female	733 (25.8)	430 (25.5)	54 (21.3)	218 (29.9)	31 (17.6)	
**Marital status**						< 0.001
Married or cohabiting	1722 (60.5)	1038 (61.5)	144 (56.7)	447 (61.4)	93 (52.8)	
Single	576 (20.2)	319 (18.9)	67 (26.4)	132 (18.1)	58 (33.0)	
Divorced or widowed	547 (19.2)	330 (19.6)	43 (16.9)	149 (20.5)	25 (14.2)	
**Ethnicity**						< 0.001
Han	2320 (81.5)	1360 (80.6)	208 (81.8)	626 (86.0)	126 (71.6)	
Zhuang	250 (8.8)	172 (10.2)	23 (9.1)	39 (5.4)	16 (9.1)	
Other	275 (9.7)	155 (9.2)	23 (9.1)	63 (8.6)	34 (19.3)	
**Education level**						0.003
Primary school or less	1572 (55.3)	949 (56.3)	123 (48.4)	420 (57.7)	80 (45.5)	
Junior middle school or more	1273 (44.7)	738 (43.7)	131 (51.6)	308 (42.3)	96 (54.5)	
**Occupation**						< 0.001
Farm	2243 (78.8)	1336 (79.2)	163 (64.2)	614 (84.3)	130 (73.9)	
None-farm	602 (21.2)	351 (20.8)	91 (35.8)	114 (15.7)	46 (26.1)	
**Transmission route**						< 0.001
Heterosexual contact	2680 (94.2)	1630 (96.6)	221 (87.0)	679 (93.3)	150 (85.2)	
Male-to-male sexual contact	64 (2.2)	21 (1.2)	28 (11.0)	0 (0.0)	15 (8.5)	
Injecting drug use	101 (3.6)	36 (2.1)	5 (2.0)	49 (6.7)	11 (6.3)	
**Year of HIV infection diagnosis**						< 0.001
2014–2015	853 (30.0)	571 (33.8)	58 (22.8)	193 (26.5)	31 (17.6)	
2016–2017	1208 (42.5)	716 (42.4)	120 (47.2)	298 (40.9)	74 (42.0)	
2018–2019	784 (27.5)	400 (23.7)	76 (29.9)	237 (32.6)	71 (40.3)	
Time from HIV diagnosis to baseline CD4+ cell count measurements, days	7 (2–23)	8 (2–25)	6 (2–18)	6 (2–19)	8 (3–23)	< 0.001

Data are presented as No. (%) or median (interquartile range). χ^2^ test for categorical data and non-parametric Wilcoxon test for continuous data.

^#^HIV-1 B/B’, CRF55_01B, CRFs and URFs.

### Effects of HIV-1 genotype on baseline CD4+ cell counts among newly diagnosed HIV patients

Table 2 presents the unadjusted and adjusted effects of HIV-1 genotype on baseline CD4+ cell counts in newly diagnosed HIV patients. Among 2845 patients, the median (IQR) baseline CD4+ cell count was 111 cells/μl (31–279), 250 cells/μl (156–377), 205 cells/μl (108–327), and 198 cells/μl (89–355) for CRF01_AE, CRF07_BC, CRF08_BC, and other genotypes, respectively. After adjusting for the potential effects of other variables in a multivariable median regression model, the coefficient of baseline CD4+ cell count was 113 (95% CI 86–140) in CRF07_BC, 80 (95% CI 65–95) in CRF08_BC, and 72 (95% CI 37–107) in the other genotypes group, which was significantly higher than that in the CRF01_AE group.

**Table 2:** Effects of HIV-1 genotype on baseline CD4+ cell counts (cells/μl) among newly diagnosed HIV patients.

HIV-1 Genotype	Patients, no. (%)	CD4+ median (IQR)	Unadjusted			Adjusted		
			Coefficient	95% CI	*P*	Coefficient	95% CI	*P*
Total	2845 (100.0)	160 (49–310)						
CRF01_AE	1687 (59.3)	111 (31–279)	0			0		
CRF07_BC	254 (8.9)	250 (156–377)	139	(117–161)	< 0.001	113	(86–140)	< 0.001
CRF08_BC	728 (25.6)	205 (108–327)	94	(73–115)	< 0.001	80	(65–95)	< 0.001
Others	176 (6.2)	198 (89–355)	87	(46–128)	< 0.001	72	(37–107)	< 0.001

Adjusted for age at diagnosis, sex, marital status, ethnicity, education level, occupation, transmission route, and year of HIV infection diagnosis.

### Effects of HIV-1 genotype on mortality before ART among newly diagnosed HIV patients

Table 3 presents the unadjusted and adjusted effects of HIV-1 genotype on mortality among newly diagnosed HIV patients in the ART-naïve cohort between 2014 and 2019. There were 451 deaths in this group and a total of 1614.2 person-years were recorded during follow-up. The overall mortality was 31.8 deaths (95% CI 29.0–34.9) per 100 person-years among the study patients. The genotype-associated mortality was 42.6 (95% CI 38.1–47.5), 16.5 (95% CI 10.6–24.5), 21.9 (95% CI 17.6–26.9), and 12.6 (95% CI 6.5–22.1) deaths per 100 person-years for patients with CRF01_AE, CRF07_BC, CRF08_BC, and other genotypes, respectively. Before ART, the hazard ratios for the association between the genotype and mortality were almost identical in both univariate and multivariate models. In multivariate analyses, compared to that in HIV patients infected with CRF01_AE, patients infected with CRF07_BC (hazard ratio, 0.55; 95% CI 0.36–0.85), CRF08_BC (hazard ratio, 0.67; 95% CI 0.52–0.85), and other genotypes (hazard ratio, 0.52; 95% CI 0.29–0.94) had a significantly lower mortality rate before ART, respectively.

**Table 3:** Effects of HIV-1 genotype on mortality before antiretroviral therapy among newly diagnosed HIV patients.

HIV-1 genotype	Patients, no	Death, no	Person-years	Deaths/100 person-years (95% CI)	HR (95% CI)	*P*	AHR (95% CI)	*P*
Total	2845	451	1614.2	31.8 (29.0–34.9)				
CRF01_AE	1687	324	760.4	42.6 (38.1–47.5)	1.00		1.00	
CRF07_BC	254	24	145.7	16.5 (10.6–24.5)	0.42 (0.28–0.63)	< 0.001	0.55 (0.36–0.85)	0.006
CRF08_BC	728	91	415.2	21.9 (17.6–26.9)	0.56 (0.44–0.71)	< 0.001	0.67 (0.52–0.85)	0.001
Others	176	12	95.0	12.6 (6.5–22.1)	0.33 (0.18–0.58)	< 0.001	0.52 (0.29–0.94)	0.030

HR (Hazard ratios) were calculated by means of both univariate and multivariate Cox regression analysis, adjusted for age at diagnosis, sex, marital status, ethnicity, education level, occupation, transmission route, year of HIV infection diagnosis, and baseline CD4+ cell count per 100 increment.

### Effects of HIV-1 genotype on mortality before ART among newly diagnosed HIV patients with baseline CD4+ cell counts < 350 cells/μl

Based on our data showing that HIV patients infected with CRF01_AE had lower baseline CD4+ cell counts (cells/μl) than those with other HIV-1 genotypes, we speculated that this might lead to a higher death rate before ART. Table 4 presents the subgroup analysis of unadjusted and adjusted effects of HIV-1 genotype on mortality among newly diagnosed HIV patients with a baseline CD4+ cell count of < 350 cells/μl in the ART-naïve cohort. A total of 424 deaths occurred in this group and 934.9 person-years were recorded during follow-up. The overall mortality was 45.3 deaths (95% CI 41.1–49.9) per 100 person-years among the study patients. The genotype-associated mortality was 57.5 (95% CI 51.1–64.4), 26.4 (95% CI 16.2–40.3), 32.0 (95% CI 26.6–40.7), and 20.9 (95% CI 10.9–36.2) deaths per 100 person-years for patients with CRF01_AE, CRF07_BC, CRF08_BC, and other genotypes, respectively. The hazard ratios for the association between the genotype and mortality were almost identical in both univariate and multivariate models. The resulting multivariate modeling showed that compared to that for HIV patients infected with CRF01_AE, patients infected with CRF07_BC (hazard ratio, 0.49; 95% CI 0.31–0.76), CRF08_BC (hazard ratio, 0.60; 95% CI 0.47–0.77), and other genotypes (hazard ratio, 0.46; 95% CI 0.26–0.82) had a significantly lower mortality rate before ART, respectively (Table 4).

**Table 4:** Effects of HIV-1 genotype on mortality before antiretroviral therapy among newly diagnosed HIV patients with baseline CD4+ cell counts < 350 cells/μl.

HIV-1 genotype	Patients, no	Death, no	Person-years	Deaths/100 person-years (95% CI)	HR (95% CI)	*P*	AHR (95% CI)	*P*
Total	2284	424	934.9	45.3 (41.1–49.9)				
CRF01_AE	1409	303	529.0	57.5 (51.1–64.4)	1.00			
CRF07_BC	176	21	78.9	26.4 (16.2–40.3)	0.49 (0.32–0.77)	0.002	0.49 (0.31–0.76)	0.002
CRF08_BC	571	88	269.6	32.0 (26.6–40.7)	0.62 (0.49–0.79)	< 0.001	0.60 (0.47–0.77)	< 0.001
Others	129	12	57.4	20.9 (10.9–36.2)	0.40 (0.22–0.71)	0.002	0.46 (0.26–0.82)	0.009

HR (Hazard ratios) were calculated by means of both univariate and multivariate Cox regression analysis, adjusted for age at diagnosis, sex, marital status, ethnicity, education level, occupation, transmission route, year of HIV infection diagnosis, and baseline CD4+ cell count per 100 increment.

### Effects of HIV-1 genotype on mortality after ART among newly diagnosed HIV patients

Table 5 presents the unadjusted and adjusted effects of HIV-1 genotype on mortality among newly diagnosed HIV patients in the ART cohort between 2014 and 2019. In this cohort, 163 deaths occurred and a total of 4301.8 person-years were recorded during follow-up. The overall mortality was 3.8 deaths (95% CI 3.2–4.4) per 100 person-years among the study patients. The genotype-associated mortality was 4.1 (95% CI 3.4–4.9), 2.2 (95% CI 0.9–4.2), 3.9 (95% CI 2.8–5.4), and 2.1 (95% CI 0.7–4.8) deaths per 100 person-years for patients with CRF01_AE, CRF07_BC, CRF08_BC, and other genotypes, respectively. The results from both univariate and multivariate analyses showed that no genotypes were significantly associated with death in this group (Table 5).

**Table 5:** Effects of HIV-1 genotype on mortality after antiretroviral therapy (ART) among newly diagnosed HIV patients.

HIV-1 Genotype	Patients, No	Death, No	Person- years	Deaths/100 person-years (95%CI)	HR (95% CI)	*P*	AHR (95% CI)	*P*
Total	2083	163	4301.8	3.8 (3.2–4.4)				
CRF01_AE	1,217	111	2699.4	4.1 (3.4–4.9)	1.00		1.00	
CRF07_BC	192	8	367.2	2.2 (0.9–4.2)	0.54 (0.26–1.10)	0.088	0.78 (0.37–1.62)	0.501
CRF08_BC	535	39	993.7	3.9 (2.8–5.4)	0.96 (0.67–1.39)	0.833	1.27 (0.86–1.87)	0.223
Others	139	5	241.5	2.1 (0.7–4.8)	0.51 (0.21–1.26)	0.144	0.95 (0.38–2.39)	0.909

HR (Hazard ratios) were calculated by means of both univariate and multivariate Cox regression analysis, adjusted for age at diagnosis, sex, marital status, ethnicity, education level, occupation, transmission route, year of HIV infection diagnosis, CD4+ cell count per 100 increment before ART, and antiretroviral regimens (grouped by zidovudine-based regimen, tenofovir-based regimen, and other regimens).

## Discussion

In the ART-naïve cohort, there were significant associations between HIV patients with CRF01_AE and lower baseline CD4+ cell count at diagnosis, as well as higher mortality, compared with CRF07_BC, CRF08_BC, and other genotypes. A previous study showed that the baseline CD4+ cell count in CRF01_AE patients was lower than that in non-CRF01_AE patients for men who have sex with men^23^. Our study further demonstrates that CRF01_AE patients have a lower CD4+ cell count than those with CRF07_BC, CRF08_BC, or other genotypes at initial diagnosis in a large population with different transmission routes. Previous studies have suggested that CRF01_AE is associated with faster AIDS progression (defined as CD4+ T-cell counts decreasing to < 200 cells/μl or < 350 cells/μl) compared to that with non-CRF01_AE^10,11^. We demonstrate for the first time that patients with CRF01_AE exhibit a significantly higher mortality compared than those with CRF07_BC, CRF08_BC, and other genotypes, in a large observational cohort study. Some studies have described that a lower baseline CD4+ cell count is associated with a risk of higher mortality compared to a higher baseline CD4+ cell count in HIV patients^24–27^. The CD4+ cell count and immune status are intermediate variables between genotypes and deaths from the perspective of epidemiological causality. To further avoid bias caused by different genotypes with heterogeneous baseline CD4+ cell counts, we performed sensitivityanalysis using a multivariate Cox model grouped by CD4+ cell count and obtained consistent results. It has been suggested that the CRF01_AE genotype is an independent risk factor for mortality in ART-naïve patients, compared with the CRF07_BC, CRF08_BC, and other genotypes. The entry of HIV into immune cells is highly dependent on the interaction of the viral envelope (R5 tropism and/or X4 tropism) with its target co-receptor (CCR5 and/or CXCR4)^28^, and an accelerated CD4+ cell count decline appears to be driven by an elevated proportion of CD4+ cells expressing CXCR4^29–31^. Researchers have shown that CRF01_AE has a higher degree of X4 tropism than non-CRF01_AE in patients infected via sexual transmission^10^.

In the ‘after ART’ cohort, our analyses revealed no significant difference in mortality among HIV patients with different genotypes, in either univariate or multivariate Cox models. In this study, the 6-year follow-up mortality of HIV patients after ART was an average of 3.8 deaths per 100 person-years, which is slightly higher than the mortality rates of some developed countries, such as England and Canada^20,21^. Previous studies have shown that there is no significant difference in immune recovery between CRF01_AE and B strains in a combined ART cohort^32^. Recently, researchers found that compared to that with CRF07_BC, CRF01_AE is associated with a lower CD4+ cell count recovery and a slower rate of immune recovery in combined ART^33^. Our study is the first to show that there is no difference in the therapeutic efficacy of combined ART among patients with CRF01_AE, CRF07_BC, CRF08_BC, and other genotypes, using death as the endpoint. Previous studies have shown that AZT exhibits a higher efficacy against viruses with R5 rather than X4 tropism. This might be explained by the fact that CCR5-expressing activated memory T cells demonstate a high efficiency in the activation of reverse transcriptase inhibitors^34–36^. Some nucleoside reverse-transcriptase inhibitors, like 3TC, are activated equally in both CCR5 and CXCR4 of target cells, and drugs do not require activation, such as the protease inhibitor ritonavir, show equal activity against both CCR5- and CXCR4-specific variants^28,36,37^.

This study showed that CRF01_AE, CRF07_BC, and CRF08_BC were the dominant HIV genotypes in Guangxi from 2014 to 2019. CRF01_AE comprised the largest proportion of the endemic strains and was composed mainly of CRF01_AE cluster 1 and cluster 2. It is worth noting that prostitutes from Vietnam have been active in the southern border area of Guangxi in recent years. CRF01_AE cluster 2, which accounts for the biggest proportion of the strains comprising the local epidemic in southern Guangxi, shows high genetic homology with the Vietnamese reference strain, according to a previous study^38^. CRF01_AE cluster 1, which is mainly prevalent in northern Guangxi, was reported to have originated in Thailand^16,39^. Our phylogenetic tree of HIV strains from the Guangxi epidemic show that these strains have formed many short branches in their evolutionary lineage, suggesting that the HIV epidemic in this area spread rapidly over a short period.

Several limitations of this study should be noted. First, censoring of information caused by loss to follow-up and ART initiation might lead to informative bias regarding the mortality rates of the ART-naïve cohort, particularly if the censoring was not random. Nevertheless, we have made an effort to measure (and include in the model) any covariates that are likely to affect the rate of censoring. Furthermore, we did not observe any differences between genotypes regarding rates of loss to follow-up and ART initiation in the ART-naïve cohort. Second, in this study, the CRF01_AE strains cluster 1 and cluster 2 comprised the bulk of the CRF01_AE infections in Guangxi, which does not cover all clusters of CRF01_AE found in China. It should be noted that we did not find any difference in mortality between CRF01_AE clusters 1 and 2 (data not shown).

However, these limitations do not invalidate our conclusions. This is the first large cohort study in a real-world setting to evaluate the effects of CRF01_AE, CRF07_BC, CRF08_BC, and other genotypes on baseline CD4+ cell counts and mortality before and after ART among newly diagnosed HIV patients. This study revealed that there is a high risk of AIDS mortality for HIV patients due to the rapid progression of CRF01_AE in ART-naïve patients, and showed that combination ART is equally efficient among HIV patients regardless of HIV-1 genotype. Our findings highlight the significance of early detection and timely treatment to reduce the risk of AIDS-related mortality.

## Materials and methods

### Study population and design

We performed an observational cohort study using data from the HIV/AIDS Comprehensive Response Information Management System (CRIMS), a Web-based real-time information collection and maintenance database for the national HIV epidemic in China, which has been described elsewhere^40–42^. Local health workers completed standardized reporting forms recording baseline information when HIV patients were newly diagnosed, and follow-up visits were conducted every 3 months before ART. For patients starting ART, follow-up visits occurred at 0.5, 1, 2, and 3 months, and then every 3 months thereafter. This study focused on patients from Guangxi, rural Southwest China. Data were collected from January 1, 2014 to April 30, 2019. The eligibility criteria were HIV-1 patients newly diagnosed between January 1, 2014 and April 30, 2019, age ≥ 18 years, baseline CD4+ cell count measurements, and willingness to participate based on written informed consent when entering CRIMS. Patients with unqualified HIV-1 sequences or the inability to link records were excluded.

Typically, baseline CD4+ cell counts would be measured immediately after patients are newly diagnosed as HIV positive. Since 2014, the national criteria for starting ART are (1) CD4+ cell count < 500 cells/μl, (2) AIDS progression to III/IV stage, or (3) willingness to receive ART, regardless of CD4+ cell counts or AIDS progression. In 2016, these guidelines were changed to offer free ART to all HIV-infected patients, regardless of CD4+ cell counts, with the provision of informed consent. According to the WHO guidelines, the current first-line combined ART regimens in China include tenofovir (TDF) or zidovudine (AZT) with lamivudine (3TC) and efavirenz or nevirapine. The second-line ART regimens include TDF or AZT with 3TC and lopinavir-ritonavir^43^.

### Procedures

Our study assessed three outcomes as follows: baseline CD4+ cell count at the time of diagnosis, mortality before ART (ART-naïve status), and mortality after ART (ART status).

The baseline characteristics and CD4+ cell counts of newly diagnosed patients in Guangxi were extracted from the CRIMS database. Patients were successively stratified by treatment status into ART-naïve and ART cohorts, according to ART initiation time. The ART-naïve cohort included HIV patients during the period spanning the date of diagnosis to the date of starting ART, or for patients who did not start ART, to the end of April 30, 2019. The ART cohort included HIV patients from the date of starting ART to April 30, 2019. To assess mortality before and after ART as a life-long process, patients who received ART after being newly diagnosed could be initially placed in the ART-naïve cohort and then moved to the ART cohort, as these groups were separated by ART initiation time.

The variables of the baseline characteristics included age at diagnosis, sex, marital status, ethnicity, education level, occupation, transmission route, year of HIV infection diagnosis, and time from diagnosis to baseline CD4+ cell count measurements. At follow-up, the following characteristics were assessed: duration of ART-naivety, date of ART initiation, initial ART regimen, duration of ART, cessation of ART, and survival status.

To analyze the effects of HIV-1 genotype on mortality before or after ART among newly diagnosed HIV patients, we used the time to death as a study endpoint. For ART-naïve patients, time zero was defined as the date of HIV diagnosis. Data were censored on the date of ART initiation or on April 30, 2019, the study endpoint. For ART patients, time zero was defined as the date of starting ART and data were censored on April 30, 2019.

### Laboratory methods and genotype analysis

HIV-1 RNA was extracted from 200 µl of stored blood samples, remaining after western blot (WB) confirmation tests, using the QIAamp Viral RNA Mini kit (Qiagen, Hilden, Germany) according to the manufacturer’s protocol. This HIV-1 RNA *pol* region encodes regions of the reverse transcriptase and protease genes (HBX2: 2253–3553), which were amplified by nested polymerase chain reaction (PCR) with commercial primers according to previously published methods^44,45^. The PCR products were purified using a QIAquick Gel Extraction Kit (Qiagen, Hilden, Germany) and sequenced by Sanger sequencing on an ABI3730xl sequencer (Life Technologies, Foster City, CA, USA) with the BigDye Terminator v3.1 kit (Life Technologies, Foster City, CA, USA). Unqualified sequences (length of nucleotide sequence < 1000 base pairs, mixed bases of nucleotide sequence > 5%) were excluded as per laboratory regulations.

The Fasttree 2.1^46^ software program was used to estimate an approximate maximum likelihood phylogenetic tree for the *pol* sequences, together with reference sequences, using a general time reversible model with g-distributed (G4) among-site rate heterogeneity. The Fasttree program made use of the Shimodaira-Hasegawa test to alternate topologies of the whole sequences and calculate the confidence of each node in phylogenetic trees. The final maximum likelihood tree was visualized using the FigTree software program, v1.4.2 (https://tree.bio.ed.ac.uk/software/figtree/).

### Statistical analysis

We analyzed differences between the various genotypes using χ^2^ tests (for categorical data) and non-parametric Wilcoxon tests (for continuous data). We estimated the median baseline CD4+ cell count by genotype among newly diagnosed HIV patients using univariate and multivariable median regression. Mortality was calculated based on Poisson distributions, in units of mortality per 100 person-years of follow-up. We performed both univariate and multivariate Cox proportional hazard models to evaluate the effects of HIV-1 genotype on mortality before and after ART among newly diagnosed HIV patients, respectively. To control for potential bias and avoid the influences of other variables, the baseline characteristics of age at diagnosis, sex, marital status, ethnicity, education level, occupation, transmission route, year of HIV infection diagnosis, and baseline CD4+ cell count were included as control variables in the multivariate median regression model and multivariate Cox proportional hazard model^23,47,48^.

A two-sided *p*-value of 0.05 or less was regarded as statistically significant. All analyses were performed using SAS 9.4 (SAS Institute, Inc., Cary, NC, USA).

### Ethics statement

This study was reviewed and approved by the institutional review board of the National Center for AIDS/STD Control and Prevention (NCAIDS), China CDC. The requirement for informed consent to participate in the study was considered unnecessary, as all patients provided informed consent when entering CRIMS at the time of diagnosis.

## Data Availability

Guangxi Center for Disease Control and Prevention permitted researchers to use the database in this study.

## References

[1] Taylor BS, Sobieszczyk ME, McCutchan FE, Hammer SM (2008). The challenge of HIV-1 subtype diversity. N. Engl. J. Med..

[2] Hemelaar J (2019). Global and regional molecular epidemiology of HIV-1, 1990–2015: A systematic review, global survey, and trend analysis. Lancet Infect. Dis..

[3] Robertson DL (2000). HIV-1 nomenclature proposal. Science.

[4] Girard M (1996). Failure of a human immunodeficiency virus type 1 (HIV-1) subtype B-derived vaccine to prevent infection of chimpanzees by an HIV-1 subtype E strain. J. Virol..

[5] Group TS (2016). Global epidemiology of drug resistance after failure of WHO recommended first-line regimens for adult HIV-1 infection: A multicentre retrospective cohort study. Lancet Infect. Dis..

[6] Kiwanuka N (2008). Effect of human immunodeficiency virus Type 1 (HIV-1) subtype on disease progression in persons from Rakai, Uganda, with incident HIV-1 infection. J. Infect. Dis..

[7] Pontiano K (2002). Effect of human immunodeficiency virus (HIV) Type 1 envelope subtypes A and D on disease progression in a large cohort of HIV-1—positive persons in Uganda. J. Infect. Dis..

[8] Vasan A (2006). Different rates of disease progression of HIV type 1 infection in Tanzania based on infecting subtype. Clin. Infect. Dis..

[9] Antiretroviral Therapy Cohort C, Canadian Observational Cohort C, Study UK, Collaboration of Observational HI (2016). Mortality of treated HIV-1 positive individuals according to viral subtype in Europe and Canada collaborative cohort analysis. AIDS.

[10] Li Y (2014). CRF01_AE subtype is associated with X4 tropism and fast HIV progression in Chinese patients infected through sexual transmission. AIDS.

[11] Chu M (2017). HIV-1 CRF01_AE strain is associated with faster HIV/AIDS progression in Jiangsu Province, China. Sci. Rep..

[12] Su Y, Liu H, Wu J, Zhu L, Wang N (2014). Distribution of HIV-1 genotypes in China: A systematic review. Zhonghua Liu Xing Bing Xue Za Zhi.

[13] Li L (2013). Different distribution of HIV-1 subtype and drug resistance were found among treatment naive individuals in Henan, Guangxi, and Yunnan province of China. PLoS ONE.

[14] Li J (2018). HIV-1 transmissions among recently infected individuals in Southwest China are predominantly derived from circulating local strains. Sci. Rep..

[15] Wang X (2017). Phylodynamics of major CRF01_AE epidemic clusters circulating in mainland of China. Sci. Rep..

[16] Feng Y (2013). The rapidly expanding CRF01_AE epidemic in China is driven by multiple lineages of HIV-1 viruses introduced in the 1990s. AIDS.

[17] Xiuling W, Jinghua H, Qiuying Z (2019). Current situation of follow-up management of the living HIV/-AIDS in Guangxi in 2017. Chin. J. AIDS STD.

[18] Lin Z, Yi C, Wang W (2019). Studying on the status of mortality of HIV/AIDS patients in Qinzhou city and its influencing factors. Chin. Health Serv. Manag..

[19] Li M (2016). Mortality among people living with HIV and AIDS in China: Implications for enhancing linkage. Sci. Rep..

[20] Croxford S (2017). Mortality and causes of death in people diagnosed with HIV in the era of highly active antiretroviral therapy compared with the general population: An analysis of a national observational cohort. Lancet Public Health.

[21] Eyawo O (2017). Changes in mortality rates and causes of death in a population-based cohort of persons living with and without HIV from 1996 to 2012. BMC Infect. Dis..

[22] Garriga C (2015). Mortality, causes of death and associated factors relate to a large HIV population-based cohort. PLoS ONE.

[23] Li X (2015). HIV-1 genetic diversity and its impact on baseline CD4+T cells and viral loads among recently infected men who have sex with men in Shanghai, China. PLoS ONE.

[24] Tang Z (2017). Effects of high CD4 cell counts on death and attrition among HIV patients receiving antiretroviral treatment: An observational cohort study. Sci. Rep..

[25] Nsanzimana S (2015). Effect of baseline CD4 cell count at linkage to HIV care and at initiation of antiretroviral therapy on mortality in HIV-positive adult patients in Rwanda: A nationwide cohort study. Lancet HIV.

[26] May MT (2016). Mortality according to CD4 count at start of combination antiretroviral therapy among HIV-infected patients followed for up to 15 years after start of treatment: Collaborative cohort study. Clin. Infect. Dis..

[27] Song A (2018). From CD4-based initiation to treating all HIV-infected adults immediately: An evidence-based meta-analysis. Front. Immunol..

[28] Schuitemaker H, vant Wout AB, Lusso P (2011). Clinical significance of HIV-1 coreceptor usage. J. Transl. Med..

[29] Berkowitz RD, Beckerman KP, Schall TJ, McCune JM (1998). CXCR4 and CCR5 expression delineates targets for HIV-1 disruption of T cell differentiation. J. Immunol..

[30] Bleul CC, Wu L, Hoxie JA, Springer TA, Mackay CR (1997). The HIV coreceptors CXCR4 and CCR5 are differentially expressed and regulated on human T lymphocytes. Proc. Natl. Acad. Sci. USA..

[31] de Roda Husman AM, Blaak H, Brouwer M, Schuitemaker H (1999). CC chemokine receptor 5 cell-surface expression in relation to CC chemokine receptor 5 genotype and the clinical course of HIV-1 infection. J. Immunol..

[32] Chow WZ (2015). Impact of HIV-1 subtype on the time to CD4+ T-cell recovery in combination antiretroviral therapy (cART)-experienced patients. PLoS ONE.

[33] Ge Z (2020). CRF01_AE and CRF01_AE cluster 4 are associated with poor immune recovery in Chinese patients under cART. Clin. Infect. Dis..

[34] Koot M (1993). Viral phenotype and T cell reactivity in human immunodeficiency virus type 1-infected asymptomatic men treated with zidovudine. J. Infect. Dis..

[35] vant Wout AB (1996). Changes in cellular virus load and zidovudine resistance of syncytium-inducing and non-syncytium-inducing human immunodeficiency virus populations under zidovudine pressure: a clonal analysis. J. Infect. Dis..

[36] van Wout AB (1997). Selective inhibition of syncytium-inducing and nonsyncytium-inducing HIV-1 variants in individuals receiving didanosine or zidovudine respectively. J. Clin. Invest..

[37] van Wout AB (1998). Efficient inhibition of both syncytium-inducing and non-syncytium-inducing wild-type HIV-1 by lamivudine in vivo. AIDS.

[38] Cheng C, Fan Y, Wu S (2009). Genetic characteristics of HIV-1 CRF01-AE strains in four provinces, southern China. Chin. J. Epidemiol..

[39] Cao Z, Yang W, Zhu Q (2019). Trend of genetic subtypes and comparison of first CD4+T cell counts in newly diagnosed HIV infections in Liuzhou, Guangxi, from 1998 to 2012. China J. Epidemiol..

[40] Zhang F (2009). Five-year outcomes of the China national free antiretroviral treatment program. Ann. Intern. Med..

[41] Zhao Y (2013). Mortality and treatment outcomes of China's National Pediatric antiretroviral therapy program. Clin. Infect. Dis..

[42] Zhang F (2011). Effect of earlier initiation of antiretroviral treatment and increased treatment coverage on HIV-related mortality in China: A national observational cohort study. Lancet Infect. Dis..

[43] National center for AIDS, STD Control and Prevention CC (2016). Manual of the national free antiretroviral treatment.

[44] Zhang J (2015). Genetic characteristics of CRF01_AE among newly diagnosed HIV-1-infected 16- to 25-year olds in 3 geographic regions of Guangxi, China. Medicine.

[45] Zhong P (2007). Genetic diversity and drug resistance of human immunodeficiency virus type 1 (HIV-1) strains circulating in Shanghai. AIDS Res. Hum. Retroviruses.

[46] Price MN, Dehal PS, Arkin AP (2009). FastTree: Computing large minimum evolution trees with profiles instead of a distance matrix. Mol. Biol. Evol..

[47] Bender R (2009). Introduction to the use of regression models in epidemiology. Methods Mol. Biol..

[48] Koenker R, Bassett G (1978). J. regression quantiles. Econometrica.

